# Understanding the social impacts of enforcement activities on illegal wildlife trade in China

**DOI:** 10.1007/s13280-021-01686-9

**Published:** 2021-12-28

**Authors:** Sifan Hu, Yu Cheng, Rong Pan, Fasheng Zou, Tien Ming Lee

**Affiliations:** 1 School of Life Sciences and State Key Laboratory of Biological Control, Sun Yat-Sen University, Guangzhou, 510275 Guangdong China; 2 School of Computer Science and Engineering, Sun Yat-Sen University, Guangzhou, 510006 Guangdong China; 3 Guangdong Key Laboratory of Animal Conservation and Resource Utilization, Guangdong Public Laboratory of Wild Animal Conservation and Utilization, Institute of Zoology, Guangdong Academy of Science, Guangzhou, 510260 Guangdong China; 4 School of Ecology, Sun Yat-Sen University, Guangzhou, 510006 Guangdong China; 5 Oxford Martin School, University of Oxford, Oxford, OX1 3BD UK

**Keywords:** Conservation, Cost-effectiveness, Online seizure news, Public whistle-blowing, Social feedback, Threatened species

## Abstract

Illegal wildlife trade enforcement is a cornerstone conservation strategy worldwide, yet we have a limited understanding on its social impacts. Using Chinese online wildlife seizure news (2003–2018), we evaluated the interactions among enforcement operations, news frequency, and social engagement (i.e., whistle-blowing) frequency. Our results showed that intensive enforcement operations, which commenced after 2012, have social impacts by increasing the frequency of all seizure news significantly by 28% [95% Confidence Interval (CI): 5%, 51%] and those via whistle-blowing by 24% [95% CI: 2%, 45%], when compared to counterfactual models where possible confounding factors were accounted for. Furthermore, we revealed the potential interaction between enforcement seizure news with and without social engagement, and the consequential social feedback process. Of the species identified from ‘whistle-blowing’ news, up to 28% are considered as high conservation priorities. Overall, we expanded our understanding of the enforcement impacts to social dimensions, which could contribute to improving the cost-effectiveness of such conservation efforts.

**Supplementary Information:**

The online version contains supplementary material available at 10.1007/s13280-021-01686-9.

## Introduction

Wildlife overexploitation and the poorly-managed nature of wildlife trade are posing direct and indirect ecological threats for biodiversity, bringing socioeconomic harm for humankind worldwide (Harris et al. 2017; Sas-Rolfes et al. 2019; Scheffers et al. 2019). In addition, pandemics resulting from disease transmission between humans and wildlife, particularly as seen in recent times, might become more regular in the future (Gibb et al. 2020). Under such an anthropogenic environmental change scenario, we need to seek solutions that require a better understanding across the natural and social sciences, which includes engagement from all sectors of society (Dhanjal-Adams et al. 2016; Reddy et al. 2017). Nevertheless, the science of behavior change and social influences has not received adequate attention in conservation science and practice (Cowling 2014; Cinner 2018; Nielsen et al. 2021).

As one of the countermeasures, law enforcement by government agencies is widely used as a fundamental strategy for managing natural resources and tackling illegal wildlife trade (IWT) (Hilborn et al. 2006; Ribeiro et al. 2019). Evaluating its evidence-based effectiveness and impacts is a priority in nature conservation (Baylis et al. 2016). Many researchers focus on evaluating the enforcement impacts on species protection (Frank and Wilcove 2019; Ribeiro et al. 2019) and potential ecological risk such as species invasion (Cardador et al. 2017). Yet, there is limited understanding of its social impact in conservation policy implementations and practices, where a progressive tightening of law enforcement could be more effective in influencing social norms and thereby compliance behavior (Keane et al. 2008; Arias 2015; Acemoglu and Jackson 2017; Salazar et al. 2019; Rizzolo 2021). As such, integrating human and social dimensions into law enforcement may promote sound conservation policymaking (Cowling 2014; Reddy et al. 2017; Sas-Rolfes et al. 2019).

Media reports play a unique role in disseminating information and public outreach (Jefferson et al. 2015; Wu et al. 2018). Online news on enforcement seizure cases provide researchers with the opportunity to explore the insights obtained from the implementation and impacts of enforcement (Siriwat and Nijman 2018) (c.f. court verdicts, which is less accessible to the public). However, researchers usually only use seizure news to reveal or monitor wildlife trafficking patterns (e.g., Patel et al. 2015; Cheng et al. 2017; Indraswari et al. 2020), and rarely on the social attributes of media reports. This might hinder the comprehensive understanding on the effectiveness of enforcement interventions and its social impacts (Walsh and O’Connor 2019; Paudel et al. 2020). For instance, there could be positive interactive effects between enforcement activities and social feedback, which may improve the cost-effectiveness of law enforcement.

Moreover, it is insufficient to rely solely on the government to deal with complex conservation problems such as IWT. Although there may be some factors that could impede the effectiveness of enforcement actions (e.g., limited resources, poverty, power dynamics and clandestine nature of the illegal activities), evidence exists that better allocation of enforcement, incorporating voluntary compliance and appropriate community and social engagement could facilitate effective natural resource management (Arias 2015; Dhanjal-Adams et al. 2016; Cooney et al. 2018; Norris et al. 2018; Salazar et al. 2019). Social engagement from non-governmental organizations or the general public through whistle-blowing on illegal activities plays a prevalent and broad role in law enforcement, particularly when laws are consistent with prevailing norms (Acemoglu and Jackson 2017). Indeed, whistle-blowing with incentives has been widely used to facilitate the timely detection of corporate fraud in business areas (Andon et al. 2018). Recent evidence in China also showed that social engagement in environment governance via whistle-blowing could have a positive and direct impact on the control of industrial air pollution (Zhang et al., 2021) and the risk of disease transmission during the outbreak of the pandemic (Chen and Chen 2020). Although whistle-blowing is also emerging in protecting wildlife as a pro-social engagement, its potential interaction with law enforcement and conservation has received limited attention (Leavitt et al. 2021).

In our study, we use online seizure news reports as a key social indicator to evaluate the potential social impacts of IWT enforcement in China. We also use public whistle-blowing data (i.e., those explicitly mentioned in the seizure news) on IWT to explore the interactive effects between law enforcement activities and social engagement. Our goal is also to understand the role of social engagement through the act of whistle-blowing in enforcement efficiency and in wildlife conservation impacts. In our context, we evaluate conservation impacts indirectly through the extent to which high conservation priority species, those of national protected status or international threatened status, are discovered from whistle-blowing. Through expanding our understanding of enforcement impacts to the social dimensions, we could provide evidence-based guidance for future strategies that optimize both enforcement efforts and social engagement to address conservation challenges.

## Theoretical framework

**Fig. 1:**
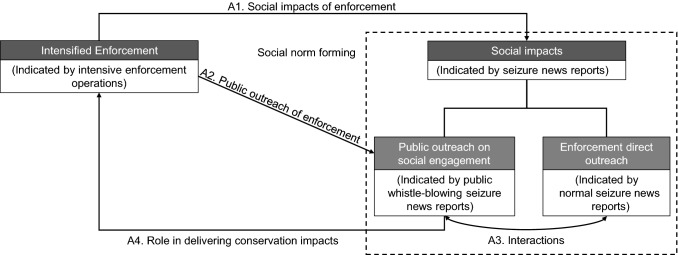
Proposed framework of the relationship between enforcement and social impact on pubic engagement. The arrows illustrate the proposed pathways with specific assumptions from intensified enforcement to the social impacts indicated by seizure news reports and social engagement via whistle-blowing seizure news reports. Law enforcement could amplify the social impacts by promoting seizure news reports (**A1**). The intensification of enforcement efforts also could have important effects on forming social norms and increasing the frequency of social engagement via whistle-blowing (**A2**). There could be a mutually reinforced interaction between enforcement and social engagement on invoking stewardship of wildlife protection. These two categories of seizure news (i.e., whistle-blowing or not) may interact in significant and complex ways (**A3**). In return, social engagement could have an important role in delivering conservation impacts (A4)

We propose a conceptual framework to explore the social impacts of enforcement and its feedback in the Chinese conservation context (Fig. 1). Specifically, we postulated that law enforcement efforts could amplify their social impacts through increasing the number of wildlife seizure reports online (A1). This process could contribute to creating social norms for proactive wildlife conservation and increasing the social intolerance for illegal wildlife activities, as well as promoting more compliance (Arias 2015; Acemoglu and Jackson 2017; Rizzolo 2021). Under this scenario, we would expect public perceptions for wildlife conservation to improve (either unconsciously or intentionally) with increasing exposure to information and the formation of social norms triggering pro-social engagement and stewardship of wildlife protection (Nolan et al. 2008; House et al. 2020). If this happens, we would also expect the intensification of enforcement efforts to increase the frequency of social engagement via whistle-blowing (A2), as well as the frequency of normal seizure news (i.e., ones that are not related to whistle-blowing). Further, we propose that these two categories of seizure news (i.e., whistle-blowing or not) may interact in complex ways (A3). Furthermore, we postulate that social engagement via whistle-blowing could also have an important role in delivering conservation impacts (A4), with respect to the numbers of species across different conservation statuses (e.g., threatened status, national protected status or international trade-regulated status).

## Materials and methods

### Case study

**Fig. 2:**
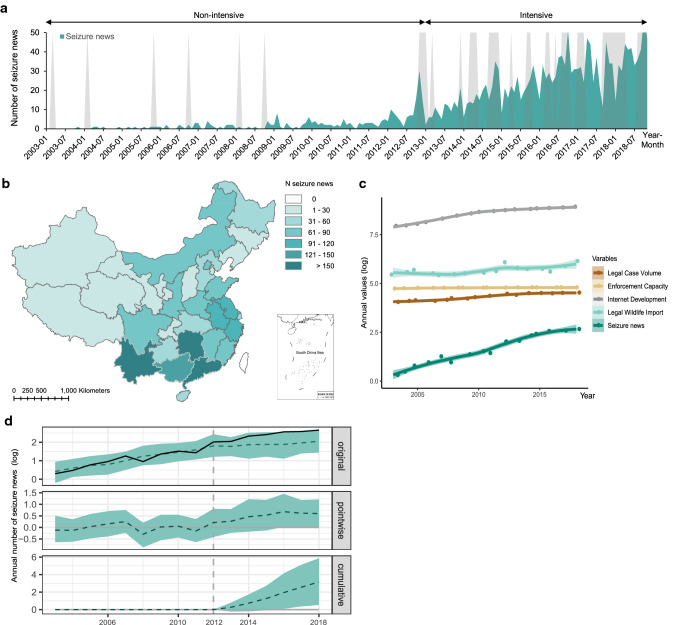
Spatial and temporal patterns of all seizure news reports and the social impacts of enforcement operations on seizure news. **a** The temporal trends of all seizure news. The gray shadows show the approximate periods of the nationwide enforcement operations on combatting illegal wildlife activities (see Table S1 of each enforcement period). The non-intensive enforcement period is between January 2003 and November 2012; the intensive period is between December 2012 and December 2018. **b** The spatial distributions of collected total seizure news reporting (n = 2020) across China. **c** The trends of seizure news and potential confounding factors on logarithmic transformation (log base 10; ‘log’) and annual level (2003–2018). **d** Visualization on the observed and modeled data about the effect of intensive nationwide enforcement operations on the number of seizure news, controlling confounding factors. The solid line shows the original data and the dashed line represents the counterfactual prediction (upper panel row). The difference between the observed and predicted data in the upper panel (the middle panel row). The sum of the values of the middle panel, reflecting a plot of the cumulative effect of the intervention (bottom panel row). The shaded area represents the 95% CI

Across the global biodiversity hotspots, China supports an exceptionally rich biodiversity (Mi et al. 2021). Facing human-induced environmental challenges due to rapid and massive economic development, the key national environmental governance strategy of ‘Ecological Civilization’ in China was initiated in November 2012 in part to promote national conservation efforts (Xiao and Zhao 2017; Mi et al. 2021). Specifically, on combatting illegal wildlife activities, nationwide enforcement operations were periodically conducted by National Forestry and Grassland Administration (NFGA) and General Administration of Customs. We collected detailed information about all nationwide enforcement operations since 2003 from the Forestry Police Section of China Forestry Yearbook (CFY 2020; Table S1). These enforcement operations were targeting any illegal activities that threatened wildlife, such as poaching, domestic and international trafficking. The temporal trends showed that intensive enforcement operations commenced around December 2012 (Fig. 2a), which coincided with the key national governance strategy of ‘Ecological Civilization’. This allowed us to explore specifically the social impacts of enforcement by comparing their patterns pre- and post-December 2012 in China. We defined post-December 2012 period as the intervention period, where intensive enforcement operations were recorded.

In recent years, whistle-blowing and the implementation of financial incentives to reward whistle-blowers has become one of the key regulatory efforts from enforcement agencies worldwide (Andon et al. 2018). For instance, the State Administration for Market Regulation and Ministry of Finance in China recently launched an initiative to encourage the public report on any illegal behavior in the markets. As such, the social engagement in environmental governance through whistle-blowing is encouraged and may be seen as a normative pro-social behavior in China (Zhang et al. 2021). Specifically, the public could call local police hotlines or use the recently available online whistle-blowing platform to report offline and online IWT activities, where the whistle-blowers are protected under the regulation of strict confidentiality in China.

### Data collection

Many public online news reports on enforcement seizure cases are available in China. Reports originating from official agencies are widely known to raise awareness among the media and public about enforcement efforts, as well as relaying legality and conservation information (i.e., a form of public outreach). Media outlets also publish related reports regularly with similar intentions. However, there is no standardized format for seizure news as they are produced by multiple sources (e.g., official agency media office or local media outlets). As such, we used purposive sampling to collect online news on seizures on Baidu News (https://news.baidu.com/). Baidu is by far the largest search engine in China, fulfilling a similar function to Google, and has been applied to IWT research (e.g., Ni et al. 2018). We conducted the search in March 2019 and the search was done backwards in time beginning with 31 December 2018 until the earliest news we could find. The final period of news reporting was therefore limited from 1st January 2003 to 31st December 2018. The key words we used are in Simplified Chinese with meanings similar to “wildlife” and “seized or smuggling or poaching”, which are widely used technical terms related to Chinese laws on wildlife protection. In this study, we only considered enforcement operations and seizure from all forms of IWT (e.g., poaching domestically, trafficking both domestically and internationally) carried out in mainland China (excluding Hong Kong, Macau or Taiwan). For each non-duplicated seizure news, the information extracted included the year of the seizure, the name of species with the quantity or weight if identified, the location of the incidents. This process was automated using Python 3.0, coupled with manual verification to confirm the consistency and accuracy of the text extraction (see the flow chart in Fig. S1).

In the seizure news, the seizure process would be reported, including whether the seizure was related to whistle-blowing. In most cases, to ensure anonymity, the news would not reveal the identity of the whistle-blowers. Instead, a general term such as “public whistle-blowing” would be commonly encountered. To assess if there is any form of social engagement in seizure cases, we would search for key words with related to “anonymous/public” and “whistle-blowing” in the seizure news so as to classify whether the seizure was related to whistle-blowing or not. In summary, we categorized seizure news in two ways: the ones that explicitly mentioned anonymous whistle-blowing as *whistle-blowing seizure news*; and the rest of the news were considered as *normal seizure news*.

We also collected possible confounding factors (2003–2018) that may have possibly driven the trend of the number of seizure news reports. They included the following: Internet Development (ID; i.e., the annual number of internet users from National Bureau of Statistics of China with the assumption that internet development would influence online media reporting; Legal Case Volume (LCV; i.e., the number of official annual criminal cases on forest and wildlife reported on CFY) with the assumption that seizure news reports are related to wildlife criminal cases; Enforcement Capacity (EC; i.e., the number of forest police officers reported on CFY annually) with the assumption that enforcement resources would affect the overall case volume; and the extent of Legal Wildlife Import (LWI; i.e., the standardized amount based on whole-organism equivalents (WOEs; Harfoot et al. 2018) of all types of imported wildlife from four terrestrial taxonomic classes that were reported by China from the Convention on International Trade in Endangered Species of Wild Fauna and Flora (CITES) database (CITES 2020) annually), where we assumed that the extent of legal wildlife imported might interact with wildlife demand, which might then have an influence on illegal wildlife activities.

### Data analysis

#### The Bayesian structural time–series model (BSTS)

Based on the known timings of the intensive nationwide enforcement operations that commenced from December 2012 (Fig. 2a, Table S1), we have pre-defined December 2012 through December 2018 (for monthly data) and years 2013–2018 (for yearly data) as the time period of intervention (post-period) for the following analyses. This particular duration was selected because the frequency of the operations has drastically increased by 4 folds, likely due to a major shift toward policies relevant to ‘Ecological Civilization’, from an average of 0.7 per year prior December 2012 to about an average of 3 per year afterward.

The Bayesian structural time–series model (BSTS) allows us to evaluate the cumulative social impact of the intensive IWT enforcement on the number of seizure news reports over time (A1) and on social engagement via whistle-blowing (A2). It could test whether the trend of the observed data is explained by the intervention, rather than by counterfactual models and confounding factors (Brodersen et al. 2015; See supplementary for equations). In our case, the BSTS models were used to test if the observed trends were impacted over time during the period of intensive nationwide enforcement operations that were carried out across China, even after accounting for potential factors.

Specifically, in the Model 1 for A1, the dependent variable was the logarithmic (base 10) number of annual seizure news reports, controlling for all possible confounding factors (ID, LCV, EC and LWI; logarithmic transformation). In the Model 2 for A2, the dependent variable was changed to the logarithmic (base 10) number of annual whistle-blowing seizure news reports, retaining all the same confounding covariates. While it is likely that we may have left out certain confounding factors, we tried to capture some of the more meaningful ecological and social influences with the best data available. Further, to evaluate the robustness of our result on A2, we conducted a monthly model (Model 3) to evaluate the impact of intensive IWT enforcement operations (after December 2012) on the number of monthly whistle-blowing seizure news reports, using normal seizure news as confounding covariate. Because both categories of reports are based on enforcement activities during the same duration, we assumed that the normal news could capture the confounding ecological and social changes and hence allowing us to evaluate the social impact of intensive enforcement.

The Model 3 was also repeated at the yearly interval for the provinces with at least 100 seizure news reports (i.e., Yunnan province: 197, Guangdong province: 193, Hunan province: 151, Guangxi autonomous region: 132, Zhejiang province: 117, Anhui: 105) to determine the impact of intensive enforcement operations from late 2012 on social engagement at each these provinces (Model SP1). Confounding factors used in Model 1 (e.g., Internet Development) were also not available at the province level. We use the provincial data at the yearly, instead of monthly, interval largely due to the low frequency of cases at the monthly interval. All BSTS models were performed with the package “CausalImpact” (Brodersen et al. 2015) in R version 4.0.2 (R Core Team 2020).

#### Granger-causality test

Given two sets of time series data, Granger-causality test allows us to test if and how normal seizure news and whistle-blowing news could have interactive effects (i.e., A3). It is a widely used method for inference on directed interactions in complex systems of many variables, with the advantages in situations where there is one or more highly autocorrelated variables (Barrett et al. 2010). It originated in the field of econometrics but has since found widespread application in many fields such as in time series inference on stochastic processes with references to energy policies (Troster et al. 2018) and cultural change (Jackson et al. 2019). For time series data, ${x}_{t}$ and ${y}_{t}$, Granger-causality is a method for determining whether one series is likely to provide more information about future values of the other than past values of itself alone (Granger 1980). In our case, we are trying to determine whether period *x*_*1*_ (the time series of the number of normal seizure news monthly) statistically provides more information about future values of *y*_*1*_ (the time series of the number of whistle-blowing news monthly) than past values of *y*_*1*_ alone; and vice versa. If so, then two time series are said to cross-correlate and interact with each other on temporal ordering. (Supplementary for equations).

To avoid spurious results, we conducted the Augmented Dickey–Fuller test (ADF) as unit root test on our data and then did the differencing transformation accordingly for stationarity, before performing our Granger-causality test. We used VARselect() function in package “vars” to select the optimal order (lag) for our Granger-causality test. In our analyses, the lag order was selected as five months, according to the information criteria of Akaike Information Criterion (AIC), Schwarz Criterion (SC), Hannan–Quinn (HQ) and Akaike's Final Prediction Error Criterion (FPE). We then repeatedly calculated the Granger-causal F-statistic for each accumulated passing period (until all 192 months were included), beginning one year ahead December 2012 (after the first 108 months) so as to adequately capture and monitor the patterns leading up to the intensive enforcement operations. We used the ‘moving’ trends of F values to demonstrate possible interactions over time at both the national-level and provincial-level. The Granger-causality tests were performed with the R package “lmtest” (Hothorn et al. 2015).

## Results

### The social impact of enforcement

A total of non-duplicate 2020 online seizure news reports (both normal and whistle-blowing news combined) was collected, where we noted increasing trends over time, with the trend particularly rising steeply beginning 2012 (Fig. 2a). The seizure news reports were from 31 provincial-level administrative divisions in mainland China, where Yunnan, Guangdong, and Hunan provinces, in descending order, recorded the greatest number of news (Fig. 2b). We found that the trend of IWT seizure news reports has significant correlations with all selected confounding factors (Internet Development, Legal Case Volume, Enforcement Capacity and Legal Wildlife Import) (All *p* < 0.05; Fig. 2c, Table S2). Nevertheless, our BSTS Model 1 unequivocally revealed that the post-2012 enforcement intervention has produced significant effects on the frequency of seizure news reports, even after accounting for all confounding factors (28% relative increase [95% CI: 5%, 51%]) (Fig. 2d, Table 1). Specifically, the logarithmic number of seizure news per year is 2.43 (cumulative value is 14.59) during the intensive enforcement operation period (post-December 2012). In contrast, and by counterfactual prediction (i.e., if the intensive enforcement operations had not taken place), we would have expected an average value of 1.90 [95% CI: 1.45, 2.34] per year (cumulative prediction value is 11.43 [95% CI: 8.72, 14.07]).

**Table 1:** Posterior inference of impacts on the number of seizure news and whistle-blowing seizure news

	Model 1 Number of seizure news (yearly, log)		Model 2 Number of whistle-blowing seizure news (yearly, log)		Model 3 Number of whistle-blowing seizure news (monthly)	
	Average	Cumulative	Average	Cumulative	Average	Cumulative
Actual	2.43	14.59	1.90	11.40	7.47	545.00
Prediction (s.d.)	1.90 (0.22)	11.43 (1.34)	1.41 (0.25)	8.46 (1.46)	6.07 (0.68)	439.15 (49.56)
95% CI	[1.45, 2.34]	[8.72, 14.07]	[0.96, 1.87]	[5.76, 11.25]	[4.67, 7.43]	[345.62, 537.84]
Absolute effect (s.d.)	0.53 (0.22)	3.16 (1.34)	0.49 (0.25)	2.94 (1.46)	1.40 (0.68)	105.85 (49.56)
95% CI	[0.09, 0.98]	[0.53, 5.87]	[0.03, 0.94]	[0.16, 5.64]	[0.10, 2.71]	[7.19, 199.40]
Relative effect (s.d.)	28% (12%)	28% (12%)	35% (18%)	35% (18%)	24% (11%)	24% (11%)
95% CI	[5%, 51%]	[5%, 51%]	[2%, 67%]	[2%, 67%]	[2%, 45%]	[2%, 45%]
Bayesian one-sided tail-area probability *p*	0.014		0.024		0.023	
Probability of a causal effect	98.6%		97.6%		97.7%	

In Model 1, the responses were the logarithmic (base 10) of the number of annual seizure news reports, controlling all the possible confounding factors (Internet development, Legal Cases Volume, Enforcement Capacity, Legal Wildlife Import; logarithmic transformation based on 10) as covariates. In Model 2, the responses were the logarithmic (base 10) of the number of annual whistle-blowing seizure news reports, controlling all the possible confounding factors as covariates. In Model 3, the responses were the number of monthly whistle-blowing seizure news reporting, controlling the monthly number of normal seizure news reporting as covariates

### The impact of enforcement on social engagement

**Fig. 3:**
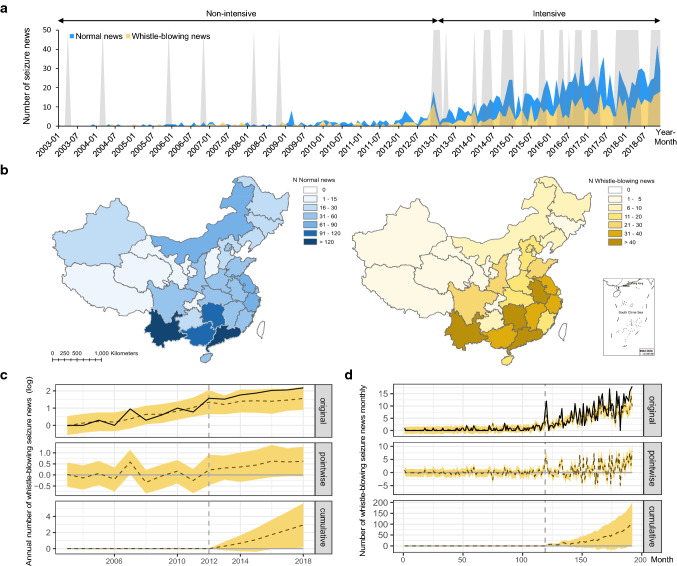
Spatial and temporal patterns of whistle-blowing seizure news and the social impact of intensive enforcement operations on whistle-blowing news. **a** The temporal trends of both normal and whistle-blowing seizure news in blue and yellow, respectively. The gray shadows show the approximate periods of the nationwide enforcement operations on combatting illegal wildlife activities. The non-intensive enforcement period is between January 2003 and November 2012; the intensive period is between December 2012 and December 2018. **b** The spatial distributions of normal news (n = 1414) and whistle-blowing news (n = 606) across China. **c** Visualization on the observed and modeled data about the effect of intensive nationwide enforcement operations on the number of whistle-blowing news yearly, controlling confounding factors. **d** Visualization on the observed and modeled data about the effect of intensive nationwide enforcement operations on the number of whistle-blowing news monthly, controlling normal news. **c** and **d** The solid line shows the original data and the dashed line represents the counterfactual prediction (upper panel row). The difference between the observed and predicted data in the upper panel (the middle panel row). The sum of the values of the middle panel, reflecting a plot of the cumulative effect of the intervention (bottom panel row). The shaded area represents the 95% CI

We identified 1414 (70.0% of all news) normal seizure news and 606 (30.0%) whistle-blowing seizure news. Both categories of seizure news increased nationwide during the observed duration (Fig. 3a and b). Moreover, our BSTS Model 2 revealed that the intensified enforcement post-2012 has a positive effect on the trend of whistle-blowing seizure news, particularly with a relative increase of 35% [95% CI: 2%, 67%], even after controlling all confounding factors (Fig. 3c, Table 1). The annual logarithmic number of whistle-blowing news reports is 1.90 during the intensive enforcement operation period (cumulative value is 11.40), compared to the expected average value of 1.41 [95% CI: 0.96, 1.87] in the absence of the intervention (cumulative prediction value is 8.46 [95% CI: 5.76, 11.25].

Even after accounting for the monthly trend of normal seizure news, our BSTS Model 3 still presents a positive and significant causal effect during the intervention period for whistle-blowing seizure news reports with 24% relative increase [2%, 45%] (Fig. 3d, Table 1). There is a monthly average of 7.47 whistle-blowing news reports during the intensive enforcement period (overall value is 545.00), compared to the expected average value of 6.07 [95% CI: 4.67, 7.43] without intensive enforcement (cumulative prediction value is 439.15 [95% CI: 345.62, 537.84]. At the provincial-level, our results (Model SP1) showed enforcement operations continue to have a stable positive effect on the number of whistle-blowing seizure news, in descending order, in the provinces of Zhejiang, Yunnan, Guangdong, and (Supplementary Result, Fig. S2, Table S3).

### Interactive effects between different types of seizure cases

**Fig. 4:**
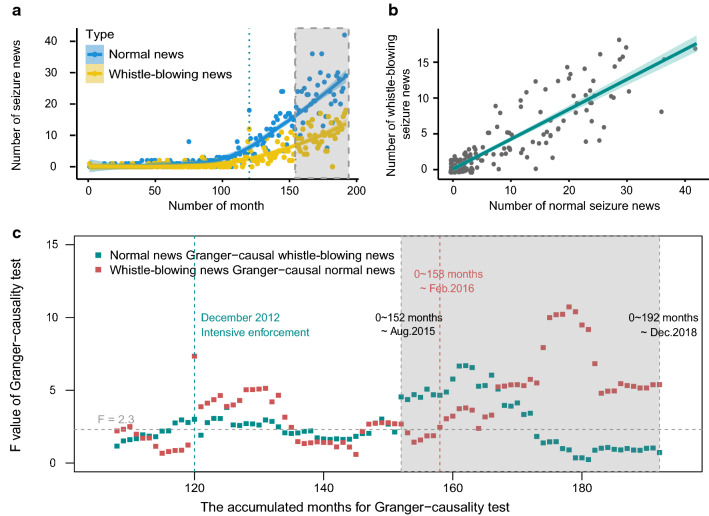
Accumulated Granger-causality statistics for the interaction between normal and whistle-blowing seizure news over time. **a** The monthly trend of normal news in blue and whistle-blowing news in yellow. The line is the regression fitted trend curve with “loess” method, with the shaded part showing the 95% CI. The gray shaded area and the dashed green line matches the time duration in plot c. **b** The correlation between the number of monthly normal news and whistle-blowing news. The line is the linear regression fitted trend, with the shade showing the 95% CI (Pearson correlation coefficient is 0.89, *p* < 0.001). **c** The F value trends of the Granger-causality test with accumulated months, beginning with the first 108 months. The Granger-causality statistics is based on differencing transformation of two time series for each accumulated period. The gray shaded area shows the period when the reciprocal Granger-causality is consistently greater than the ‘minimum’ significance level of F value (≥ 2.3, Lag order = 5)

The Granger-causality analysis demonstrated that the differencing-transformed normal seizure news interacted with whistle-blowing news and the interaction appears to be reinforced for a certain period of time (Fig. 4; correlated temporal normal and whistle-blowing news trends: r = 0.89, *p* < 0.001). We observed a fluctuation in the accumulated granger F-statistics trends in either direction before August 2015, but the positive effect, where the normal seizure news significantly ‘granger-caused’ whistle-blowing news began to appear after the intensive IWT enforcement had commenced for 32 months (cumulative 152 months; F_Normal152_ = 4.55, *p*_Normal152_ < 0.001) (Fig. 4c). This relationship was maintained at a stable and significant level for another 20 months until April 2017 (F_Normal172_ = 3.43, *p*_Normal172_ = 0.006). As this relationship began to weaken, another positive feedback, where whistle-blowing news ‘granger-caused’ normal news (in the reverse direction), started to emerge after the intensive enforcement had commenced for 38 months in February 2016 (cumulative 158 months; F_Whistle-blowing158_ = 2.46, *p*_Whistle-blowing158_ = 0.036). This new positive feedback attained a highly significant level after another eight months in November 2016 (F_Whistle-blowing167_ = 5.23, *p*_Whistle-blowing167_ < 0.001) and was maintained until the end of the study duration.

While the interactive effects emerged at the national-level, variations in the onset of their timing and patterns were seen across different provinces. For example, while the effect that normal seizure news ‘granger-caused’ whistle-blowing news is apparent in Yunnan, Guangdong, Guangxi, and Zhejiang provinces, this relationship appeared the earliest in Guangzhou (only about a year after the intensified enforcement operations; Fig. S3).

### Conservation impact from whistle-blowing

**Fig. 5:**
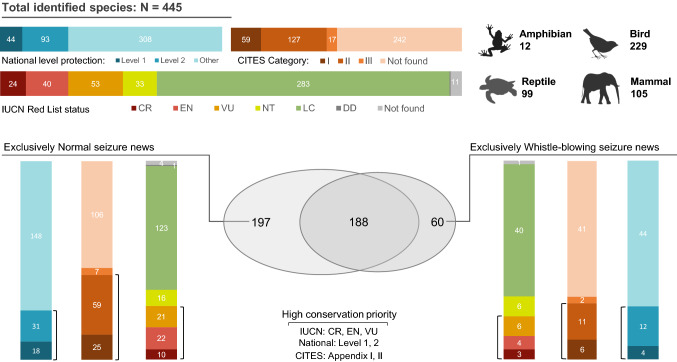
Summary of the number of identified species and the species threatened status. The blue gradient percentage bar-plots present the percentages of national-level protection of China (1989–2020). The red gradient percentage bar-plots presents the percentages of Convention on the International Trade in Endangered Species of Wild Fauna and Flora categories (CITES 2019). The colorful gradient percentage bar-plots presents the percentages of International Union for Conservation of Nature Red List on threatened status (IUCN 2019; CR: Critically Endangered, EN: Endangered, VU: Vulnerable, NT: Near Threatened, LC: Least Concern, DD: Data Deficient)

In total, we identified 445 species: 12 amphibians; 99 reptiles; 229 birds; and 105 mammals (Table S4), most of which (78%) are native species according to the list of China’s vertebrate (Jiang et al. 2016). The species data revealed 60 species were exclusively detected by whistle-blowing news and 197 species were exclusively uncovered by normal news; and 188 species appeared in both seizure news categories (Fig. 5). Among these 445 species, a total of 137 species (31%) are on the original national list of protected species of China (1989–2020) (44 and 93 species on level one and level two, respectively). According to the International Union for Conservation of Nature Red List of Threatened Species (IUCN Red List) database (IUCN 2019), the seized species consisted of 24 Critically Endangered species (CR, 5%), 40 Endangered species (EN, 9%), 53 Vulnerable species (VU, 12%), 33 Near Threatened species (NT, 7%) and 283 Least Concern species (LC, 64%). Based on the CITES database (CITES 2019), the seized species included 59 species listed on Appendix I (13%) that are threatened with extinction and prohibited from international commercial trade, 127 species on Appendix II (29%) that are not necessarily threatened with extinction yet but must be regulated from international trade, and 17 species on Appendix III (4%) that may be regulated from international trade at the request of a Party and would require the cooperation of other countries; more than half (54%) are non-CITES regulated species.

We consider species of high conservation priorities as those which have either local (i.e., China national list level one or two) and/or international (i.e., IUCN Red List CR, EN, or VU, and CITES Appendix I and II) conservation statuses. For the 60 species reported only in whistle-blowing news, 22% (IUCN), 27% (National List) and 28% (CITES) are high conservation priorities; for the 197 species unique to normal seizure news, 27% (IUCN), 25% (National List), and 43% (CITES) are of high conservation concerns. Comparing species exclusively reported in either normal or whistle-blowing news, we did not find significant differences in the proportions among the three conservation or trade regulation statuses (χ^2^_IUCN_ = 0.41, χ^2^_National Level_ = 0.01, χ^2^_CITES_ = 3.37) (Fig. 5; Table 2).

**Table 2:** Proportions of each threatened status in both normal seizure news and whistle-blowing seizure news group

Categories			Total	All		Exclusively		Conservation concern			
								All		Exclusively	
				Normal	Whistle-blowing	Normal	Whistle-blowing	Normal	Whistle-blowing	Normal	Whistle-blowing
			n = 445 (%)	n = 385 (%)	n = 248 (%)	n = 197 (%)	n = 60 (%)	n = 385 (%)	n = 248 (%)	n = 197 (%)	n = 60 (%)
IUCN Red List status	Threatened	CR	24 (5%)	21 (6%)	14 (6%)	10 (5%)	3 (5%)	104 (27%)	64 (26%)	53 (27%)	13 (22%)
		EN	40 (9%)	36 (9%)	18 (7%)	22 (11%)	4 (7%)				
		VU	53 (12%)	47 (12%)	32 (13%)	21 (11%)	6 (10%)				
	Low risk	NT	33 (7%)	27 (7%)	17 (7%)	16 (8%)	6 (10%)	281 (73%)	184 (74%)	144 (73%)	47 (78%)
		LC	283 (64%)	243 (63%)	160 (65%)	123 (62%)	40 (67%)				
	Other	DD	1 (0%)	1 (0%)	–	1 (1%)	–				
		Not found	11 (2%)	10 (3%)	7 (3%)	4 (2%)	1 (1%)				
National protected level		Level 1	44 (10%)	40 (10%)	26 (10%)	18 (9%)	4 (7%)	121 (31%)	88 (35%)	49 (25%)	16 (27%)
		Level 2	93 (21%)	81 (21%)	62 (25%)	31 (16%)	12 (20%)				
		Other	308 (69%)	264 (69%)	160 (65%)	148 (75%)	44 (73%)	264 (69%)	160 (65%)	148 (75%)	44 (73%)
CITES Category		I	59 (13%)	53 (14%)	34 (14%)	25 (13%)	6 (10%)	169 (44%)	102 (41%)	84 (43%)	17 (28%)
		II	127 (29%)	116 (30%)	68 (27%)	59 (30%)	11 (18%)				
		III	17 (4%)	13 (3%)	14 (6%)	7 (3%)	2 (3%)	216 (56%)	146 (59%)	113 (57%)	43 (72%)
		Not found	242 (54%)	203 (53%)	132 (53%)	106 (54%)	41 (68%)				

Chi-Squared Test was conducted on conservation concern for exclusively Normal and Whistle-blowing groups: IUCN Red List status: χ^2^ = 0.41, df = 1, *p* = 0.520; National protected level: χ^2^ = 0.01, df = 2, *p* = 0.912; CITES Appendix: χ^2^ = 3.369, df = 3, *p* = 0.066

## Discussion

Our research explores the links between IWT enforcement and its social impacts, especially on social engagement, to advance our understanding on the interactive effects between policy and social dimensions when addressing wildlife conservation challenges (Fig. 1). Indeed, enforcement is not only key to combatting illegal threats directly, but it could also have social impacts through the resulting seizure news reports, which in turn could encourage social engagement by whistle-blowing, particularly crucial under limited enforcement resources (Figs. 2, 3). It is possible that the social impact of enforcement on promoting news reports could have an influence in the formation of social norms, thus aligning the social perceived norms with the existing laws (Arias 2015; Acemoglu and Jackson 2017). In addition, the public may become partly influenced by the growing information exposure, albeit unconsciously, in the online news environment (Nolan et al. 2008). When social engagement via whistle-blowing on wrongdoing becomes widespread and normatively appropriate, such behavior could develop into social norms thereby promoting pro-social behavior (Bergquist et al. 2019; House et al. 2020). Moreover, our finding suggests that ‘top-down’ effect (e.g., law enforcement promotes social norms and engagement) might interact and lead to ‘bottom-up’ feedback (e.g., social concerns and engagement facilitate enforcement efforts) (Fig. 4). Future conservation efforts on strengthening law enforcement efforts could incorporate our understanding of the social impact and its positive feedback, by considering the interplay between law enforcement and social norms (Acemoglu and Jackson 2017; Rizzolo 2021). Moreover, drawing from our insight, future design of behavior change strategies should encourage social engagement, where it is essential to increase self-efficacy about how to act and strengthen the social support regarding socially appropriate norms via concrete public outreach information (Klöckner 2013; Bergquist et al. 2019).

Notably, we observed that the surge in law enforcement operations in late 2012 appeared to coincide with China’s commitment to the ‘Ecological Civilization’ ideology first proposed in November 2012 (Xiao and Zhao 2017). Meanwhile, many efforts have been carried out to mainstream biodiversity conservation and improve public conservation awareness in China (Zhang and Yin 2014; Olmedo et al. 2020). As such, it would be important to further explore in-depth the interaction between enforcement and social impacts. Media reports could be utilized as one of the main approaches to continuously monitor this interaction, as well as to strengthen social feedback (Wu et al. 2018). With China’s goal to fulfill the ecological vision (Mi et al. 2021), enforcement agencies could leverage the growing trend of social impacts to deliver conservation outcomes. Having the right conditions such as appropriate social norms could also maximize the benefits of social engagement in conservation, as well as environmental issues (Zhang et al. 2021). Furthermore, monitoring the interaction between enforcement and social engagement, and exploring feedback mechanism in various regions and context could guide the implementation on where and when public outreach efforts could be enhanced for positive social impacts as we face growing environmental issues (e.g., biodiversity conservation).

For the conservation impacts, we noticed that whistle-blowing and normal seizure news in China both contribute to a substantial percentage of high conservation priority species (22%-43%), though the absolute numbers are much lower for the former than the latter. The low numbers and proportion (17 out of 60) from whistle-blowing news might be due to the highly clandestine nature of the illegal wildlife activities of high conservation priority species, or the rudimentary conservation knowledge that the public are simply alerting the authorities to any perceived illicit activities. Besides, there is a higher probability for the public to encounter low conservation priority species, which are relatively more common. This evidence would be beneficial for future conservation intervention designs, aiming at enhancing social engagement in conservation governance. For instance, increasing a cadre of volunteers or professionals with related training and capacity might contribute to more targeted whistle-blowing (e.g., bird-watchers on illegal bird trade). Improving conservation knowledge about wildlife could potentially increase public whistle-blowing knowledge on species with high conservation priority (Keane et al. 2011; Green 2016). As such, limited resources for enforcement make targeted public engagement even more crucial, where wasting resources and enforcement inefficiency should be avoided. Therefore, further efforts are needed to empower the public and encourage ‘trained’ volunteers to focus on high conservation priority species, in addition to certain species of low conservation need that may require immediate conservation action, to raise and improve overall enforcement efficiency and conservation successes.

Enforcement agencies could also lay the foundation for social engagement to help meet the challenges posed by the increasingly concealed and organized illegal IWT (Sas-Rolfes et al. 2019), such as leveraging citizen science on intelligence gathering platforms with digital technologies (e.g., Walsh and O’Connor 2019; Chauhan and Gallacher 2021). For example, in China, one of the online whistle-blowing platforms is on one of the most popular social media platforms (i.e., WeChat) through the official account called “Tencent For The Planet”. The platform was jointly launched in 2015 by Tencent and several international conservation organizations (e.g., International Fund for Animal Welfare), with support from the main governmental agency responsible for conservation in China (i.e., NFGA). Optimizing the whistle-blowing platforms could either promote enforcement efficiency or increase public perceived behavior control, which could in turn motivate more people to participate (Klöckner 2013; Chauhan and Gallacher 2021). Future research could use the platforms to evaluate the effectiveness of social engagement and explore the trigger mechanism of public whistle-blowing to unpack the decision-making processes in conservation, as these details are almost always omitted in the news. Notably, while new technologies to improve social engagement have been developed in criminology (e.g., Walsh and O’Connor 2019; Chauhan and Gallacher 2021), implementing them in the IWT context and across various different societies might be challenging. Related regulatory efforts will need to carefully consider the social context, local norms and acceptability of such actions to minimize unnecessary side effects. Furthermore, proper legal and administrative protocols must be in place and the enforcement agencies must ensure that informants are sufficiently protected from being victims of violent retaliation.

Admittedly, although we attempted to extract and include as much online seizure news reports as possible, there may still be some potential sampling issues. Nevertheless, we were able to use the publicly accessible ‘independent’ Chinese criminal court verdict data from ‘Openlaw’ (http://openlaw.cn/) to cross-validate the extent of temporal (r = 0.97, *p* < 0.001) and geographical congruence (r = 0.79, *p* < 0.001) in our online news sample, giving us confidence in our results (Supplementary Methods, Fig. S4). Additionally, although anonymous whistle-blowing were explicitly mentioned in news, there remains the possibility of inaccurate estimation of such news due to various reasons. As such, we partially evaluated the classification accuracy of the whistle-blowing news using court verdicts. Using two key provinces as examples, we manually and thoroughly matched 78 of 197 news reports (about 40%) with corresponding court verdicts from Yunnan province and 56 of 193 news (about 30% match rate) from Guangdong province. The low rate may be because not all seizure in news reached the criminal courts (i.e., those under Administrative Penalty Law in China where the cases are generally less severe than crimes), and relatively fewer verdicts from before 2013 were recorded in ‘Openlaw’. Despite this, we still uncover a relatively high accuracy classification on whistle-blowing news of the matched data (Yunnan: 0.81 [95%CI: 0.70, 0.89]; Guangdong: 0.79 [95%CI: 0.66, 0.88]) and higher proportion of whistle-blowing in news (Table S5). Moreover, although the interpolated and extrapolated sampling coverages on seized species indicated the detected species sampling is nearly complete (estimated at 93.6% for all seizure news) (Fig. S5), our seizure news likely also suffer from taxonomic bias. Similar to previous study, there appears to be more underreporting in amphibians and reptiles than birds and mammals (Thomas-Walters and Raihani 2017). This potentially limits our comprehensive understanding on the impact of social engagement via whistle-blowing, especially on other groups such as plants (Sas-Rolfes et al. 2019), which are also heavily trafficked in China and globally (e.g., orchids, Hinsley et al. 2018).

## Conclusion

In this paper, our evidence-based research evaluates one of the most understudied yet vital areas of the interactions between organized enforcement efforts and social engagement in conservation context. Our results illustrate the positive social impact of nationwide enforcement operations on news reporting and pro-social engagement (i.e., whistle-blowing) in China. This form of social engagement could produce some conservation impacts, where species of high conservation priorities have been seized. Our findings, as seen across China and a handful of provinces, provide data-driven guidance for enforcement strategies to the governments to optimize enforcement efforts through promoting social impact via social engagement. We conclude that leveraging our understanding of the interactions between IWT enforcement operations and social engagement could assist governments to achieve the mutual empowerment and social amplifications required to scale-up cost-effective efforts to address IWT and other conservation challenges.

## Supplementary Information

Below is the link to the electronic supplementary material.

Supplementary file1 (PDF 1862 KB)
