# Supplementary material for: Understanding the social impacts of enforcement activities on illegal wildlife trade in China
Source: Ambio. 2021 Dec 28;51(7):1643–57. doi: 10.1007/s13280-021-01686-9 (PMC9110577; doi:10.1007/s13280-021-01686-9)
Supplement: Supplementary file 1 — Supplementary file1 (PDF 1862 KB) [file 13280_2021_1686_MOESM1_ESM.pdf]

Ambio

Supplementary Information

Title: **Understanding the social impacts of enforcement activities on illegal wildlife trade in China**

This PDF file includes:

Supplementary Methods

Supplementary Results

Supplementary Figures: Figure S1 to Figure S5

Supplementary Tables: Table S1 to Table S5

Supplementary References

Supplementary Methods

Data mining and text extraction

We used purposive sampling to collect online news reports on wildlife seizure in China. A purposeful search was conducted between January and March 2019 on Baidu News (https://news.baidu.com/) using key Chinese characters with meaning similar to "wildlife" and "seized or smuggling or poaching" (i.e., Chinese words "野生动物" with "查获", "截获", "走私", "贩卖", "非法猎捕", "非法狩猎"), which are widely used technical terms related to Chinese laws on wildlife protection. We used the key words in Simplified Chinese, considering the nationwide enforcement operations are carried out only in mainland China (excluding Hong Kong, Macau or Taiwan). Each search period was a month, beginning with December 2018, and the search was done backwards in time until the earliest news we could find. The final search was therefore limited to seizure news from January 2003 to December 2018.

After the news URL links were crawled (n = 5118) from the web, we downloaded contents from each link and extracted the main text. Then, text processing algorithms were implemented to segment the Chinese characters and phrases using Python 3.0. Based on the accessibility of the news URL link and the relevance of the news contents, we first filtered the news using computer algorithms, followed by manual proofreading. Later, based on the availability of information on wildlife and locality, we proceeding with the second stage news filtering leaving behind 3441 news. We further removed 1365 news that are not seizure news where there is no quantity data of wildlife (e.g., news of wildlife campaigns).

We repeatedly checked for duplication and news filtering protocol to remove unrelated news, including those without specific locality, topics about zoo and farms and those unofficial investigations by journalists, resulting in 2137 news left. We then further excluded 10 seizure news where Hong Kong, Macau or Taiwan was implicated either as an origin, a transit, or a destination. Based on another round of duplication check, we finalized a set of 2020 news for further analyses. For each unique and non-duplicated seizure news, the information extracted included the year of the seizure, the media source, the name of species with the quantity or weight and the location of the incidents (see the flow chart in Fig. S1).

Data analysis with equations

The Bayesian structural time–series model (BSTS)

The BSTS model allows us to evaluate the posterior inference of impacts and estimate the cumulative effect of the interventions on the number of seizure news reports over time. It could be defined as follows (Brodersen et al., 2015):

$$y_t = \mu_t + \tau_t + \beta^T x_t + \varepsilon_t$$

$$\mu_t = \mu_{t-1} + \delta_t + v_t$$

$$\delta_t = \delta_{t-1} + v_t$$

The equation includes all the components that explain the number of the observed data y_t. The first component, μ_t, is the value of the trend at time t. The δ_t component is the expected increase in μ, between t and $t+1$, presents a random walk pattern. So, it can be thought of as the *slope* at time t. The vector x_t is a set of potential control series candidate

to be predictive of the response. All covariates are assumed to be contemporaneous. The error terms ε_t follow independent Gaussian random noises, N (0, σ_t^2).

Granger-causality Test

Given two sets of time series data, x_t and y_t, granger-causality is a method for determining whether one series is likely to provide more information about future values of the other than past values of itself alone (Granger, 1969; Granger, 1980). It is based on linear prediction theory for detecting causal relations among multiple linear time series, which is usually implemented via autoregressive (AR) modelling of multivariate time series. This is accomplished by taking different lags of one series and using that to model the change in the second series. It creates two models which predict y, one with only past values of y_t (Ω), and the other with past values of y_t and x_t (π). The models are given below where k is the number of lags in the time series:

Let $\Omega = y_t = \beta_0 + \beta_1 y_{t-1} + \ldots + \beta_k y_{t-k} + \varepsilon$;

$\pi = y_t = \beta_0 + \beta_1 y_{t-1} + \ldots + \beta_k y_{t-k} + \alpha_1 x_{t-1} + \ldots + \alpha_k x_{t-k} + \varepsilon$.

The residual sums of squared errors are then compared and a test is used to determine whether the nested model (Ω) is adequate to explain the future values of y_t or if the full model (π) is better. The F-test is calculated to test the following null and alternate hypotheses:

H_0: $\alpha_i = 0$ for each i of the element [1, k];

H_1: $\alpha_i \neq 0$ for at least 1 i of the element [1, k].

We examined provincial trends from only six provinces where there are at least 100 or more seizure news reports for similar trends (i.e., Yunnan, Guangdong, Hunan, Guangxi, Zhejiang and Anhui). While the number of months selected as the first granger causality tests depended on the number of news to ensure both normal seizure news and whistle-blowing news at least around 30 (our minimum sample size), the maximum observed is 118 months (January 2003 – December 2012) for the first granger causality tests, when the intensive enforcement commenced. Again, the Augmented Dickey–Fuller test (ADF) as unit root test was also conducted for stationarity before performing granger-causality test to avoid spurious regression results. The two time-series in six provincial-level regions revealed stationarity and hence there is no need for further differencing transformation. The optimal lag order was selected as 10 for Yunnan, 2 for Guangdong, 9 for Hunan, 7 for Guangxi, and 5 for both Zhejiang and Anhui.

Sample bias and coverage

To evaluate the reliability or potential temporal (i.e., year) and geographical (i.e., province) biases that our seizure news data may have, we collected the openly accessible court verdicts from Openlaw (http://openlaw.cn/) as a corroborating source. Based on the cases classification scheme of Criminal Law in China, four types of wildlife crime were identified: wildlife trafficking, wildlife poaching, wildlife smuggling and illegal wildlife hunting. We searched for all non-duplicate court verdict cases under these four categories from 2003 to 2018 and retrieved 8846 court verdicts in total (wildlife trafficking: 2606, wildlife poaching: 1092, wildlife smuggling: 388, illegal wildlife hunting: 4760). Although

some court verdicts may also contain wildlife crimes, we only count those where the main crime is related to wildlife. We first use principal component analysis to reduce the dimensions of the four types of wildlife crimes at the yearly and provincial levels. Pearson correlation was used to assess 1) the consistency of the temporal annual trend between the number of seizure news and principal component 1 (PC1) of court verdicts, and 2) the congruence of geographic distribution between the number of seizure news and PC1 of each province.

Furthermore, although anonymous whistle-blowing were explicitly mentioned in some news reports, there may still be a possibility for inaccurate estimation due to various reasons. To evaluate the potential classification accuracy of our sample, we also used court verdicts for cross-validation. However, the limitations of the court verdicts from Openlaw include 1) only cases of Criminal Law (more severe in nature) but not those of Administrative Penalty Law (less severe) hence potentially underestimation the overall number of cases in China; and 2) relatively fewer verdicts were available before 2014 when the platform established. Using seizure news from Yunnan as an example, and after manually evaluating the contents, we were able to successfully match 78 (about 40%) news reports to the court verdicts (a total of 1519 court verdicts on wildlife offences were observed in Yunnan from 2003 to 2020 to account for delay in court prosecution). Using seizure news from Guangdong as another example, we were able to successfully match 56 (about 30%) news reports to the court verdicts (a total of 904 court verdicts on wildlife offences were observed in Guangdong from 2003 to 2020). We analysed the matched data

with a confusion matrix, using confusionMatrix() function in package “caret” and using mcc() function in package “mltools”, specifically for the Matthews correlation coefficient in our binary classification evaluation. In detail, we selected the whistle-blowing seizure news group as the “Positive” Class. A set of appropriate statistics were computed which include the following (Chicco and Jurman 2020):

Overall accuracy = $\frac{\mathrm{TP} + \mathrm{TN}}{\mathrm{TP} + \mathrm{TN} + \mathrm{FP} + \mathrm{FN}}$;

Balanced Accuracy = $\frac{\mathrm{Sensitivity} + \mathrm{Specificity}}{2}$;

Sensitivity (True Positive Rate) = $\frac{\mathrm{TP}}{\mathrm{TP} + \mathrm{FN}}$;

Specificity (True Negative Rate) = $\frac{\mathrm{TN}}{\mathrm{TN} + \mathrm{FP}}$;

Positive Predictive Value = $\frac{\mathrm{TP}}{\mathrm{TP} + \mathrm{FP}}$;

Negative Predictive Value = $\frac{\mathrm{TN}}{\mathrm{TN} + \mathrm{FN}}$;

Matthews correlation coefficient (MCC) = $\frac{\mathrm{TP} * \mathrm{TN} - \mathrm{FP} * \mathrm{FN}}{\sqrt{(\mathrm{TP} + \mathrm{FP})(\mathrm{TP} + \mathrm{FN})(\mathrm{TN} + \mathrm{FP})(\mathrm{TN} + \mathrm{FN})}}$;

(TP: true positive, TN: true negative, FP: false positive, FN: false negative).

To evaluate the sample completeness and coverage, sample-size, coverage-based rarefaction and extrapolation metrics were used to provide the estimates of total species richness and ‘sampling’ completeness for all species and seizure news types (Chao and Jost, 2012; Chao et al., 2014). We performed the sample completeness curves (95% CI) in package “iNEXT” (Chao et al. 2014), which reveals sample completeness for a given sample size. This curve also provides an estimate of the sample size needed to achieve a certain degree of completeness.

Supplementary Results

The impact of enforcement on social engagement at the provincial level

At the provincial-level, BSTS models generally show the intensive and nationwide enforcement operations beginning from late 2012 in China producing stable effects on the number of whistle-blowing news (Model SP1) in Yunnan, Guangdong and Zhejiang (Fig S2, Table S6).

In Yunnan province (152 normal news and 45 whistle-blowing news), the positive and significant causal effect is observed during the intervention period on whistle-blowing news with a relative increase of 73% [95% CI: 5%, 132%]. The number of whistle-blowing news per year is 6.7 (sum of 40.0) during the intensive enforcement operation period (post-2012), compared to the expected average value of 3.9 [95% CI: 1.6, 6.5] (sum of 23.1 [95% CI: 9.4, 38.8]) in the absence of the intervention and controlling for trend of normal seizure news. In Guangdong province (146 normal news and 47 whistle-blowing news), the number of whistle-blowing news per year is 6.7 (sum of 40.0) during the intensive enforcement operation period (post-2012). If the intensive enforcement operations had not taken place, we would have expected average value of 4.2 [95% CI: 2.4, 6.2] (sum of 25.1 [95% CI: 14.3, 37.1]) after controlling for normal news in terms of absolute numbers. The positive and significant causal effect is observed during the intervention period on whistle-blowing news with a relative increase of 59% [95% CI: 12%, 102%]. In Zhejiang province (82 normal news and 35 whistle-blowing news), the positive and significant causal effect is observed during the intervention period on whistle-blowing cases with a relative increase of

112% [95% CI: 22%, 198%] after controlling for normal news. The number of whistle-blowing news per year is 5.5 (sum of 33.0) during the intensive enforcement operation period (post-2012), compared to the expected average value of 2.6 [95% CI: 0.4, 4.9] (sum of 15.5 [95% CI: 2.3, 29.6]) in the absence of the intervention.

In Hunan province (100 normal news and 51 whistle-blowing news), the intervention appears to have caused a negative effect the number of whistle-blowing news (relative effect: -31% [95% CI: -64%, 1%]), while this effect fluctuates and is not significant. Moreover, although a positive effect is observed in Guangxi province (95 normal news and 37 whistle-blowing news) (relative effect: 11% [95% CI: -33%, 53%]) and in Anhui province (53 normal news and 52 whistle-blowing news) (relative effect: 35% [95% CI: -14%, 82%]) after controlling for the trends of normal news, the effects fluctuates and are both not significant.

Interactive effects between different types of seizure cases at the provincial level

At the provincial-level, granger causality tests for determining the interactive effects between the trends of normal news and whistle-blowing news could provide regional reference to decision-making of public outreach efforts in each province. Generally, stable effect that normal news could have influence on whistle-blowing news were detected for a certain duration after accumulated periods in Yunnan, Guangdong, Guangxi and Zhejiang, while this effect was seen earliest in Guangdong. In particular, the significant granger-causality was observed in Guangdong about one year after the intensive enforcement

commenced in November 2013. Furthermore, the granger-causal relationship in Yunnan appeared slightly later than Guangdong in March 2015, followed by Zhejiang (observed in March 2016) and Guangxi (appeared in February 2017). In contrast, in Hunan and Anhui, the granger-causality of normal news on whistle-blowing news was strong at the beginning then decrease steadily (Fig. S3).

Specifically, in Guangdong province, we detected a stable effect where the normal seizure news significantly predicted whistle-blowing news after the intensive enforcement commenced for just 11 months in November 2013 (cumulative 131 months; $F_{130} = 1.73$, $p_{130} = 0.182$; $F_{131} = 4.31$, $p_{131} = 0.015$). Plotting the granger statistics over accumulated months, we tracked the degree of the granger-causality remains at a stable level (F value have a bit of fluctuation from 4 to 6) in Guangdong. Pooling all data in Yunnan province and plotting the granger statistics over accumulated months, we tracked the influence of normal news on whistle-blowing news which fluctuated then increased with time. A significant effect was detected when the normal seizure news granger-caused whistle-blowing news in March 2015, which is after the intensive enforcement commenced for 27 months (cumulative 147 months; $F_{146} = 1.94$, $p_{146} = 0.047$; $F_{147} = 2.71$, $p_{147} = 0.005$). With time, the degree of the granger causality increased and reached a relative high level where F value approximately exceeded 8). In Zhejiang province, we failed to observe the significant effect of normal news on whistle-blowing news until in March 2016 (cumulative 159 months). A stable and significant influence appeared and maintained afterwards ($F_{158} = 1.68$, $p_{158} = 0.143$; $F_{159} = 4.94$, $p_{159} < 0.001$, after the intensive enforcement commenced for

39 months. In Guangxi province, we tracked the temporal ordering effect of normal news on whistle-blowing news increasing then stabilizing significantly from February 2017 (cumulative 170 months), after the intensive enforcement commenced for 50 months (F_{169} = 1.42, p_{169} = 0.200; F_{170} = 2.16, p_{170} = 0.041). With time, the degree of the interactive effect increases until a stable effect was attained (F value approximately equals to 6) in August 2017 (cumulative 176 months), which is about 56 months after the intensive enforcement operations began.

In Hunan province, we detected a stable effect where the normal seizure news significantly 'granger-caused' whistle-blowing news all the time. The influence of normal seizure news reports on whistle-blowing news decreased first, then stabilized at a significant level (F value approximately equal to 10), and decreased and reached another stable level (F value approximately equals to 4). Similarly, in Anhui province, we also detected a stable effect all the time where the normal seizure news significantly predicted whistle-blowing news. Such effect stabilized at a significant level first (F value approximately equal to 10), and decreased and reached another stable level (F value approximately equals to 6).

Supplementary figures and tables

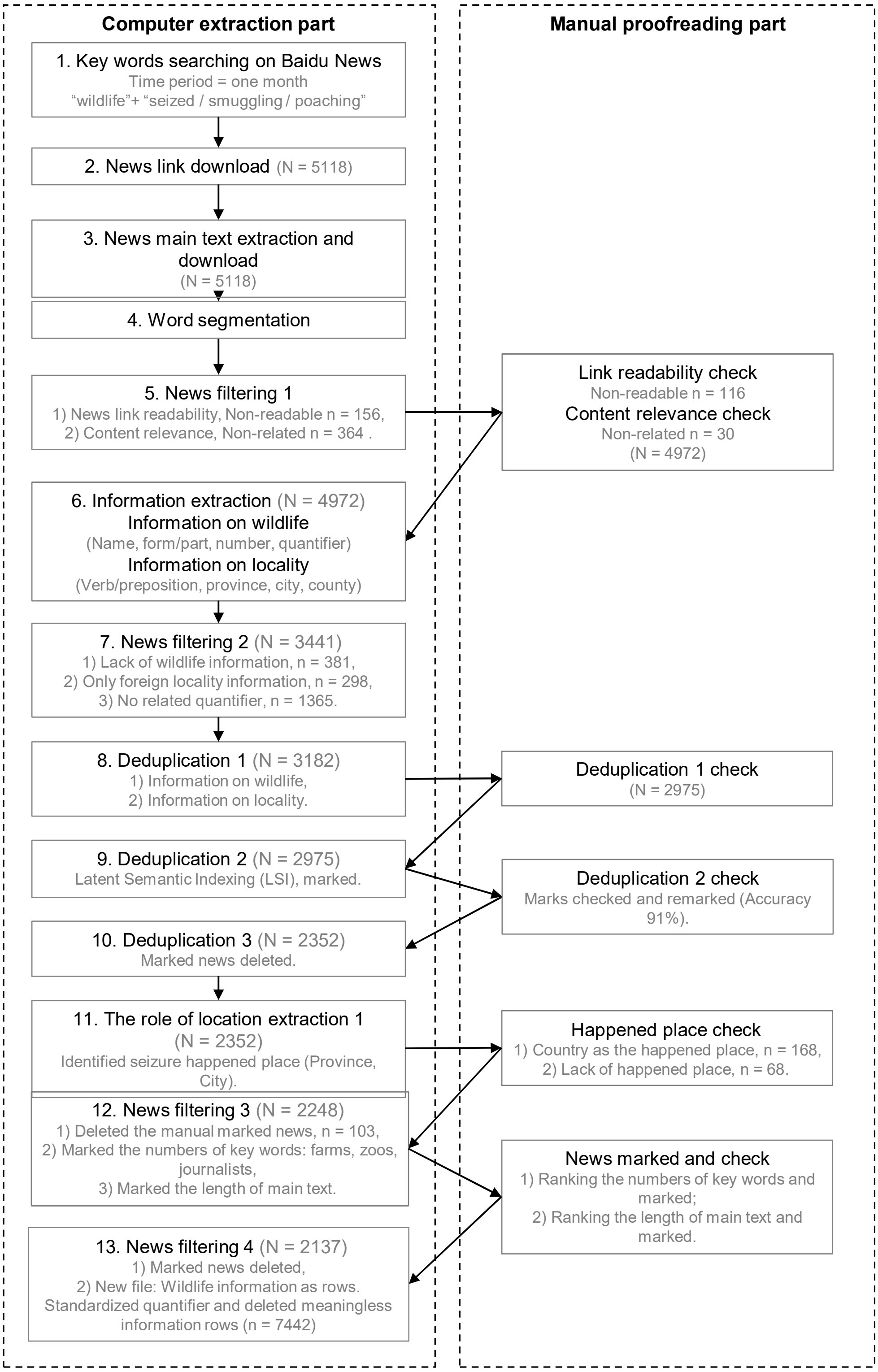

Figure S1. The flow chart of the data collection and information extraction *(To be continued in following page)*.

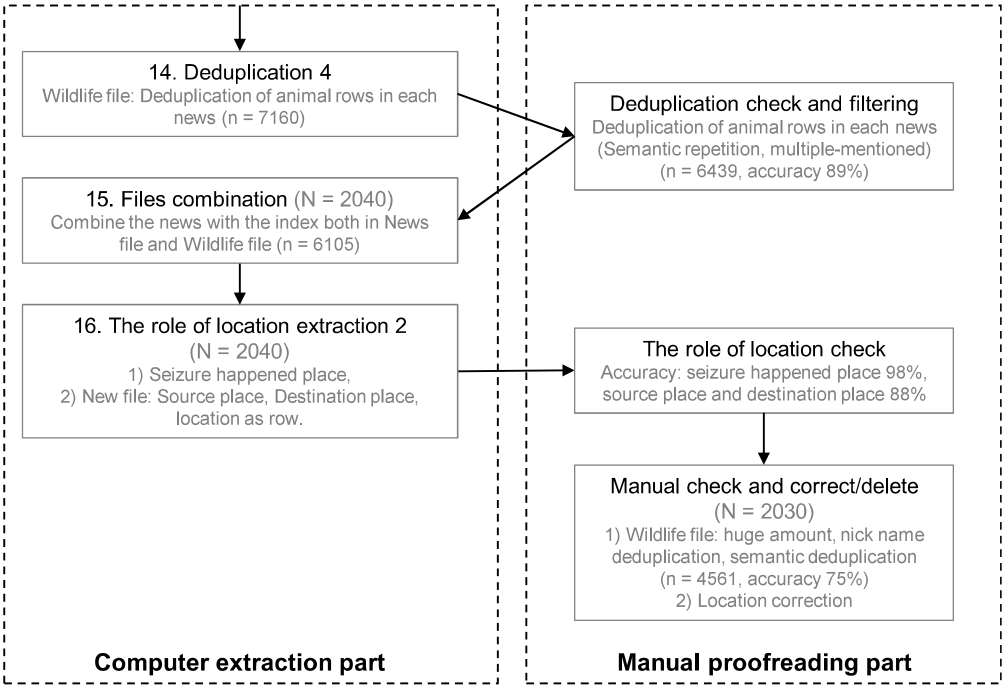

Figure S1*(Continued)*. The flow chart of the data collection and information extraction.

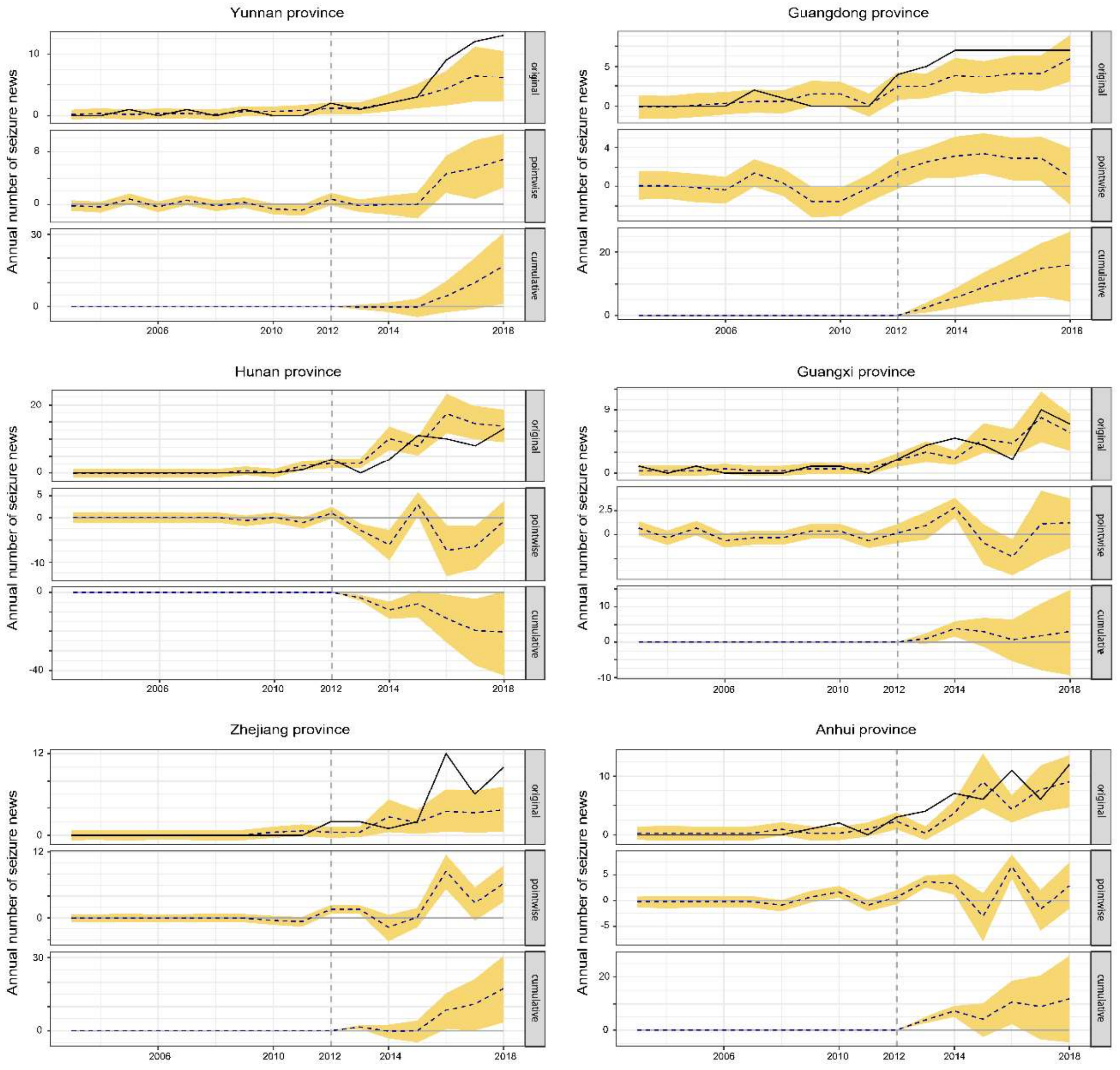

Figure S2. Visualization on the observed and modeled data about the effect of intensive enforcement operations on the annual number of whistle-blowing seizure news at provincial-level. The number of normal seizure news reports is controlled as covariate in the models. Each set of seizure news group in each province contains three panels. Upper panel: the solid line shows the original data and the dotted line represents the counterfactual prediction. The middle panel: the difference between the observed and predicted data in panel one. The lower panel: sums the values of the middle panel, resulting in a plot of the cumulative effect of the intervention. The shade presents the 95% CI.

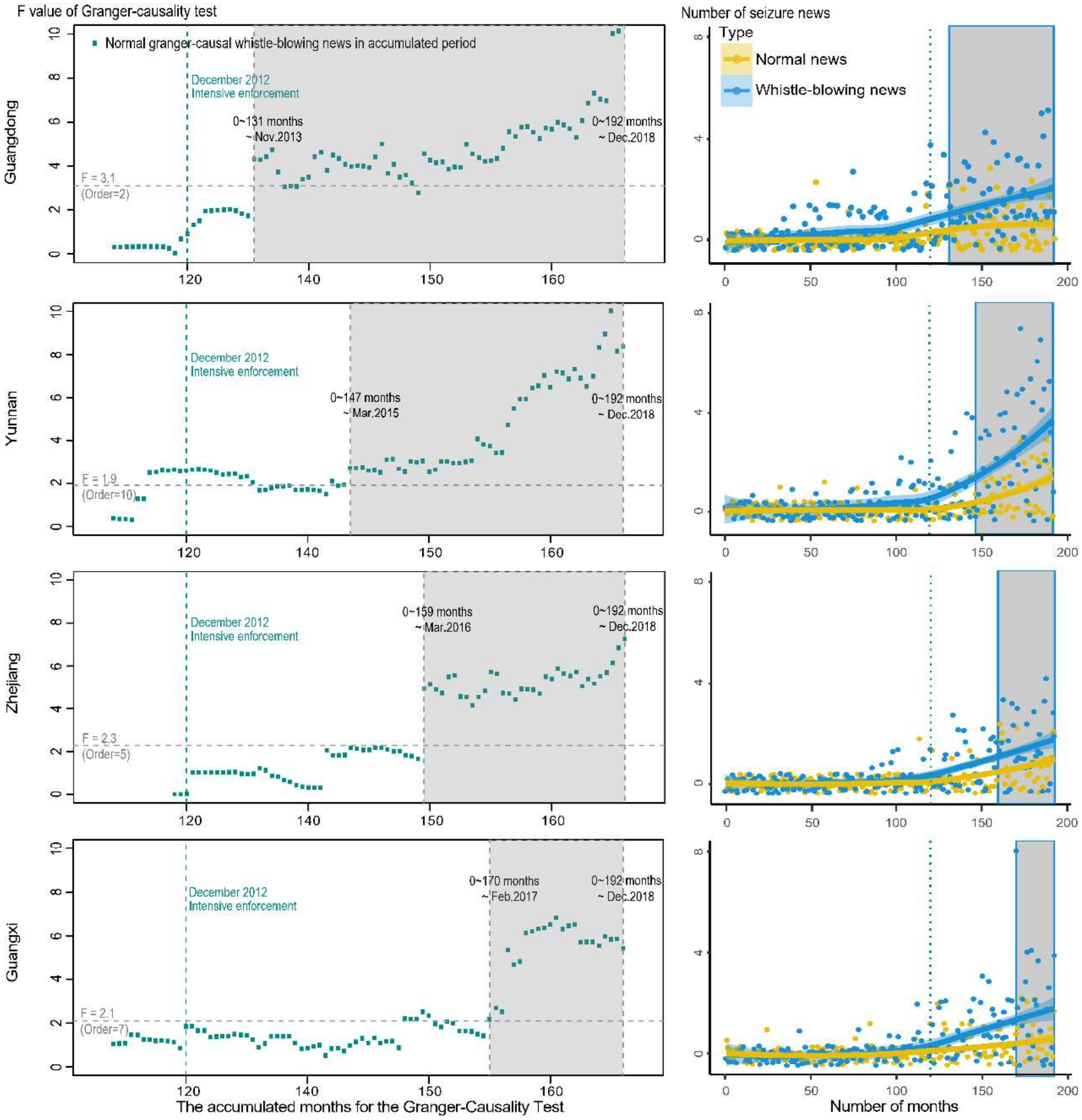

Figure S3. The level of interaction over time at provincial-level. Left: The F value trends of granger-causality test with accumulated months. Each green point presents the granger-causality statistic from the number of normal seizure news to whistle-blowing seizure news. The grey shaded area shows the duration where significant granger-causality statistics are sustained. The horizontal dotted line shows the significance level based on F value. The green dotted line shows the period of intensive nationwide enforcement operations started (December 2012). **Right:** The monthly trend of normal news in blue and whistle-blowing news in yellow. The line is the regression “lowess” fitted trend curve, with the shaded part showing the 95% CI. The grey shaded area is to match the period in Left with the corresponding granger-causality tests. *(To be continued in following page)*.

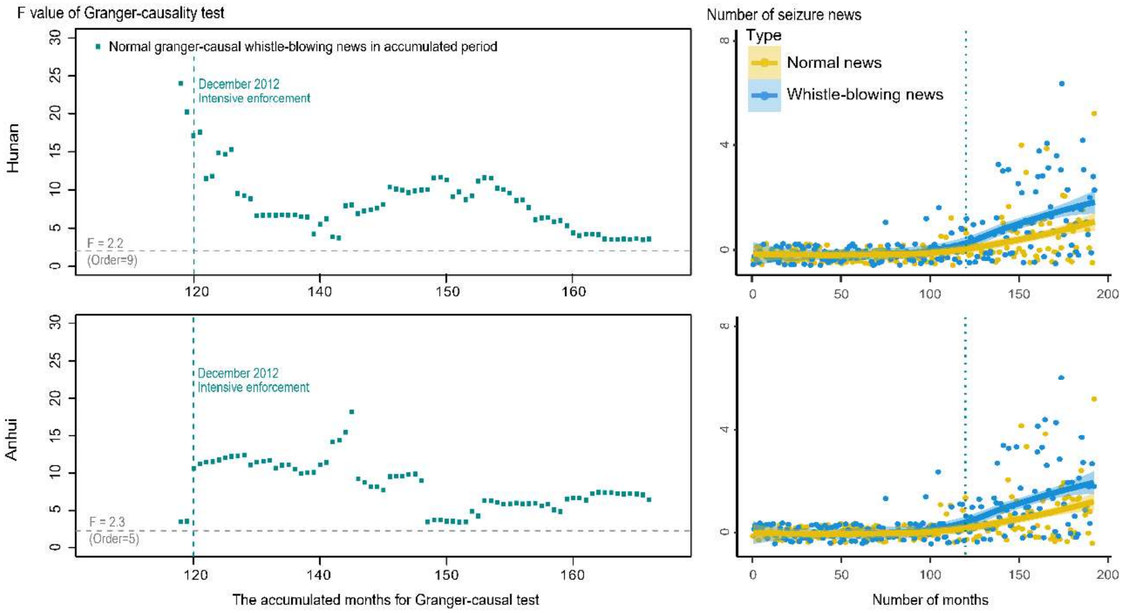

Figure S3 *(Continued)* **The level of interaction over time at provincial-level. Left:** The F value trends of granger-causality test with accumulated months. Each green point presents the granger-causality statistic from the number of normal seizure news to whistle-blowing seizure news. The grey shaded area shows the duration where significant granger-causality statistics are sustained. The horizontal dotted line shows the significance level based on F value. The green dotted line shows the period of intensive nationwide enforcement operations started (December 2012). **Right:** The monthly trend of normal news in blue and whistle-blowing news in yellow. The line is the regression "lowess" fitted trend curve, with the shaded part showing the 95% CI. The grey shaded area is to match the period in Left with the corresponding granger-causality tests.

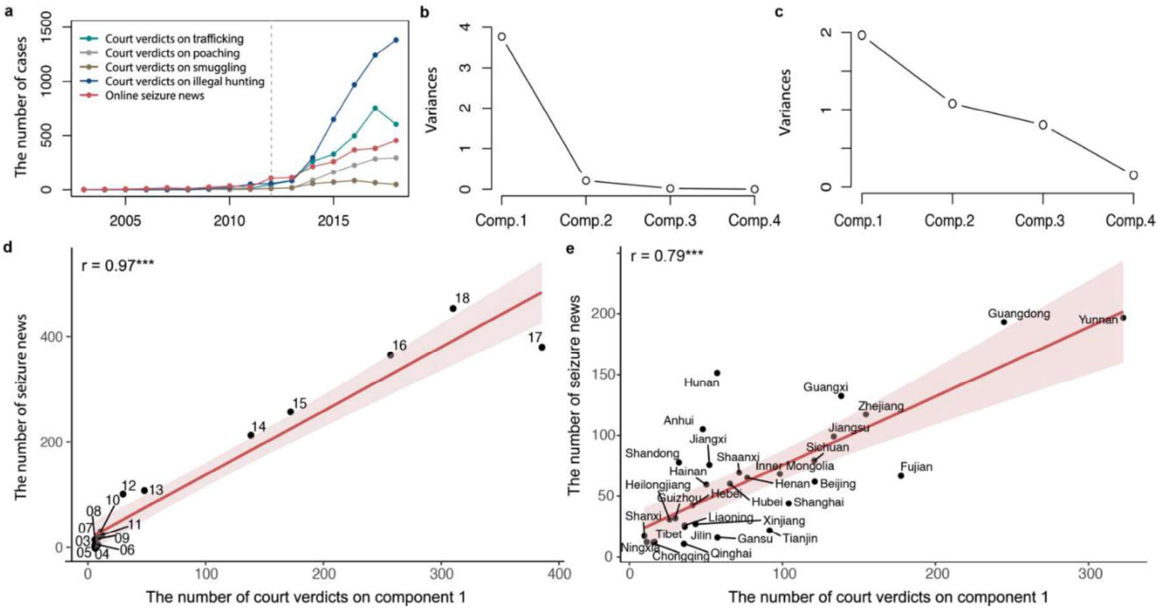

Figure S4. The comparison of temporal trends and spatial distribution between collected seizure news and online court verdicts from *Openlaw*. a) The annual trends of collected seizure news reports and court verdicts on four types of wildlife crime. The dotted line is year 2012. **b)** The scree plot of principal component analysis on the annual number of four-type wildlife crimes court verdicts. **c)** The scree plot of principal component analysis on the number of four-type wildlife crimes court verdicts in each province. **d)** The correlation between the number of seizure news and component 1 (PC 1) of court verdicts on annual level. Pearson correlation r = 0.97, $p < 0.001$. **e)** The correlation between the number of seizure news and the component 1 (PC 1) of court verdicts on provincial level. Pearson correlation r = 0.79, $p < 0.001$. The line is the linear regression fitted trend, with the shade of 95%CI range.

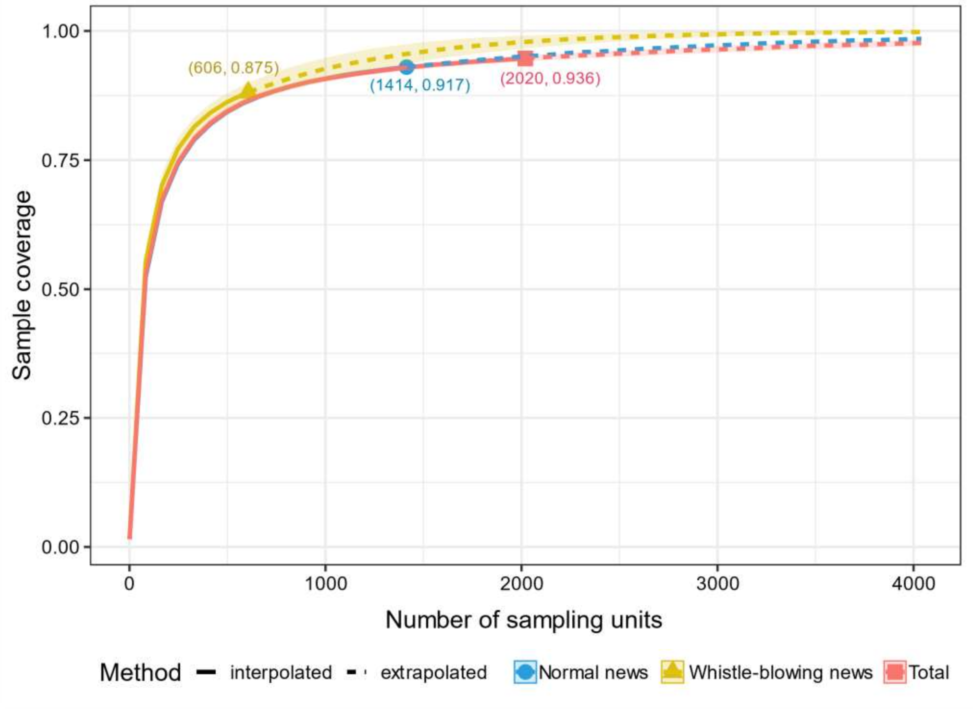

Figure S5. Plot of sample coverage for all the collected online seizure news reports covering species detected. The solid lines are the rarefied samples. The dashed lines are the extrapolated samples. The shades present 95%CI range. The curve was extrapolated up to a doubling of the total reference sample size (n = 4040). Reference samples are denoted by solid dots. Numbers in the parentheses represent the number of sampling units and the sample coverage, respectively.

Table S1. The list of the nationwide enforcement operations from 2003 to 2018 on combatting illegal wildlife activities.

Name of the nationwide enforcement operation[1, 2, 3]	Beginning date (Month/Date/Year)	Ending date (Month/Date/Year)
Operation Spring-Thunder (春雷行动)	04/10/2003	04/19/2003
Migratory Birds II (候鸟二号)	03/21/2004	03/31/2004
Winter Crackdown (冬季严打)	12/01/2005	01/31/2006
Operation Green-Shield (绿盾行动)	11/01/2006	12/31/2006
Operation Flying-Eagle (飞鹰行动)	03/20/2008	05/31/2008
Migratory Birds III (候鸟三号)	11/10/2008	12/10/2008
Operation on Combatting Illegal Wildlife Activities (打击破坏野生动物资源违法犯罪专项行动)	11/27/2012	12/31/2012
Operation Cobra I (眼镜蛇一号行动)*	01/06/2013	02/05/2013
Operation Sky-Net 2013 (2013 天网行动)	04/01/2013	04/30/2013
Operation on Combatting Illegal Wildlife Activities (打击破坏野生动物资源违法犯罪专项行动)	08/15/2013	12/28/2013
Operation Cobra II (眼镜蛇二号行动)*	12/30/2013	01/26/2014
Operation Sky-Net 2014 (2014 天网行动)	04/01/2014	06/30/2014
Operation Sharp-Sword 2014 (2014 利剑行动)	10/10/2014	01/31/2015
Operation Cobra III (眼镜蛇三号行动)*	05/04/2015	05/27/2015
Operation Thunder (雷霆行动)	09/30/2015	11/30/2015
Operation on Combatting Illegal Wildlife Activities (打击破坏野生动物资源违法犯罪专项行动)	04/20/2016	05/20/2016
Operation Clean-Net (净网行动)	07/01/2016	07/15/2016
Operation on Combatting Illegal Forest-related activities (打击非法占用林地等涉林违法犯罪专项行动)	09/01/2016	11/01/2016
Operation on Migratory Birds Protection (保护候鸟专项行动)#	10/18/2016	11/27/2016
Operation Sharp-Sword 2017 (2017 利剑行动)	02/17/2017	04/30/2017
Operation on Migratory Protection (护飞行动)#	10/26/2017	11/30/2017
Operation Hurricane I (飓风 1 号专项行动)	12/11/2017	01/31/2018
Operation Thunder 2018 (雷霆 2018)	02/01/2018	03/31/2018
Operation Spring-Thunder 2018 (春雷 2018)	04/01/2018	05/31/2018
Operation Green-Sword 2018 (绿剑 2018)	09/01/2018	12/10/2018

Notes:

1. The information of specific nationwide enforcement operation is collected from the Forestry Police Section of China Forestry Yearbook (http://navi.cnki.net/KNavi/YearbookDetail?pcode=CYFD&pykm=YGFDS&bh=).
2. The Chinese names are the official name from the Forestry Police Section of China Forestry Yearbook, the English names are non-official translation by the authors.
3. The nationwide enforcement operation by Customs that non-specifically targeting wildlife are not in the list.

* presents the international joint operation for tackling wildlife smuggling.

\# presents the special operation for migration birds' conservation.

Table S2. The correlations among the annual trend of seizure news reports and the confounding factors.

	S	LCV	EC	ID	LWI
**Seizure news (S)**	1.00				
**Legal Case Volume (LCV)**	0.96***	1.00			
**Enforcement Capacity (EC)**	0.91***	0.89***	1.00		
**Internet Development (ID)**	0.94***	0.95***	0.93***	1.00	
**Legal Wildlife Import (LWI)**	0.68**	0.73**	0.56*	0.59*	1.00

Note:

1. The annual trends were the logarithmic (base 10) values.
2. Significance: * $p < 0.05$, ** $p < 0.01$, *** $p < 0.001$.

Table S3. The posterior inference of impacts on the number of whistle-blowing seizure news reports yearly on provincial level.

Province		Model SP1 Number of whistle-blowing seizure news (yearly)	
		Average	Cumulative
**Yunnan (n = 197)** (152 normal and 45 whistle-blowing seizure news)	**Actual**	6.7	40.0
	**Prediction (s.d.)**	3.9 (1.2)	23.1 (7.3)
	**95% CI**	[1.6, 6.5]	[9.4, 38.8]
	**Absolute effect (s.d.)**	2.8 (1.2)	16.9 (7.3)
	**95% CI**	[0.2, 5.1]	[1.2, 30.6]
	**Relative effect (s.d.)**	**73% (32%)**	**73% (32%)**
	**95% CI**	**[5%, 132%]**	**[5%, 132%]**
	**Bayesian one-sided tail-area probability *p***	0.020	
	**Probability of a causal effect**	97.97%	
**Guangdong (n = 193)** (146 normal and 47 whistle-blowing seizure news)	**Actual**	6.7	40.0
	**Prediction (s.d.)**	4.2 (1.0)	25.1 (5.8)
	**95% CI**	[2.4, 6.2]	[14.3, 37.1]
	**Absolute effect (s.d.)**	2.5 (1.0)	14.9 (5.8)
	**95% CI**	[0.5, 4.3]	[3.0, 25.7]
	**Relative effect (s.d.)**	**59% (23%)**	**59% (23%)**
	**95% CI**	**[12%, 102%]**	**[12%, 102%]**
	**Bayesian one-sided tail-area probability *p***	0.006	
	**Probability of a causal effect**	99.40%	
**Hunan (n = 151)** (100 normal and 51 whistle-blowing seizure news)	**Actual**	7.7	46.0
	**Prediction (s.d.)**	11.0 (1.8)	66.0 (11.0)
	**95% CI**	[7.6, 15.0]	[45.5, 89.0]
	**Absolute effect (s.d.)**	-3.4 (1.8)	-20.4 (11.0)
	**95% CI**	[-7.1, 0.1]	[-42.6, 0.4]
	**Relative effect (s.d.)**	-31% (17%)	31% (17%)
	**95% CI**	[-64%, 0.7%]	[-64%, 0.7%]
	**Bayesian one-sided tail-area probability *p***	0.031	
	**Probability of a causal effect**	96.89%	

(To be continued)

Table S3 *(Continued)*. **The posterior inference of impacts on the number of whistle-blowing seizure news reports yearly on provincial level.**

Province		Model SP1<br>The number of whistle-blowing seizure news (yearly)	
		Average	Cumulative
	**Actual**	5.2	31.0
	**Prediction (s.d.)**	4.7 (1.0)	28.0 (6.0)
**Guangxi**	**95% CI**	[2.7, 6.7]	[16.3, 40.3]
**(n = 132)**	**Absolute effect (s.d.)**	0.5 (1.0)	3.0 (6.0)
	**95% CI**	[-1.6, 2.5]	[-9.3, 14.7]
(95 normal and 37	**Relative effect (s.d.)**	11% (22%)	11% (22%)
whistle-blowing	**95% CI**	[-33%, 53%]	[-33%, 53%]
seizure news)	**Bayesian one-sided tail-area probability *p***	0.314	
	**Probability of a causal effect**	68.60%	
	**Actual**	5.5	33.0
	**Prediction (s.d.)**	2.6 (1.1)	15.5 (6.9)
**Zhejiang**	**95% CI**	[0.4, 4.9]	[2.3, 29.6]
**(n = 117)**	**Absolute effect (s.d.)**	2.9 (1.1)	17.5 (6.9)
	**95% CI**	[0.6, 5.1]	[3.4, 30.7]
(82 normal and	**Relative effect (s.d.)**	**112% (44%)**	**112% (44%)**
whistle-blowing	**95% CI**	**[22%, 198%]**	**[22%, 198%]**
seizure news)	**Bayesian one-sided tail-area probability *p***	0.011	
	**Probability of a causal effect**	98.88%	
	**Actual**	7.7	46.0
	**Prediction (s.d.)**	5.7 (1.4)	34.1 (8.3)
**Anhui**	**95% CI**	[3.0, 8.4]	[18.0, 50.6]
**(n = 105)**	**Absolute effect (s.d.)**	2.0 (1.4)	12.0 (8.3)
	**95% CI**	[-0.8, 4.6]	[-4.6, 27.9]
(53 normal and 52	**Relative effect (s.d.)**	35% (24%)	35% (24%)
whistle-blowing	**95% CI**	[-14%, 82%]	[-14%, 82%]
seizure news)	**Bayesian one-sided tail-area probability *p***	0.080	
	**Probability of a causal effect**	92.08%	

Note: In model SP1, the number of normal seizure news in each year are included as covariate.

Table S4. Taxonomic information of the species from the seizure news (n = 2020).

Class	Number of news	Number of taxonomic groups identified		
	News with class included[1]	Species[2]	Family[3]	Order
**Mammal**	1009	105	36	11
**Bird**	929	229	53	22
**Reptile**	539	99	26	4
**Amphibian**	178	12	8	2
**Total**	/	445	123	39

Notes:

1. One seizure news could include more than one taxonomic group on class level. The overall number would be larger than the actual number of seizure news collected because of the overlapping.
2. There are 1476 seizure news that the information of taxonomic groups could be identified on species level.
3. Taxonomic groups on family level might cover more beyond the number of species, since some common name in news only can reach family level.

Table S5. Confusion Matrix of matched seizure news with corresponding online court verdicts in a) Yunnan province (N = 78) and b) Guangdong province (N=56).

**a) Confusion Matrix for Yunnan**			
		**Online Court verdicts ('ground truth' data)**	
		Normal	Whistle-blowing
**Seizure news (or observed data)**	Normal	57	1
	Whistle-blowing	14	6
**Statistics ("Positive" Class: Whistle-blowing)**			
Overall accuracy		0.81	
95% CI		(0.70, 0.89)	
Balanced accuracy		0.83	
Sensitivity (True Positive Rate)		0.86	
Specificity (True Negative Rate)		0.80	
Positive predictive value		0.30	
Negative predictive value		0.98	
Matthews correlation coefficient (MCC)		0.43	

**b) Confusion Matrix for Guangdong**			
		**Online court verdicts ('ground truth' data)**	
		Normal	Whistle-blowing
**Seizure news (or observed data)**	Normal	40	0
	Whistle-blowing	12	4
**Statistics ("Positive" Class: Whistle-blowing)**			
Overall accuracy		0.79	
95% CI		(0.66, 0.88)	
Balanced accuracy		0.88	
Sensitivity (True Positive Rate)		1.00	
Specificity (True Negative Rate)		0.77	
Positive predictive value		0.25	
Negative predictive value		1.00	
Matthews correlation coefficient (MCC)		0.44	

Note:

Evaluation statistics follow the formulae from Chicco & Jurman, 2020.

Supplementary references

Brodersen, K. H., F. Gallusser, J. Koehler, N. Remy, and S. L. Scott. 2015. Inferring causal impact using bayesian structural time-series models. *Annals of Applied Statistics* 9: 247–274. doi:10.1214/14-AOAS788.

Chao, A., and L. Jost. 2012. Coverage-based rarefaction and extrapolation: Standardizing samples by completeness rather than size. *Ecology* 93: 2533–2547. doi:10.1890/11-1952.1.

Chao, A., N. J. Gotelli, T. C. Hsieh, E. L. Sander, K. H. Ma, R. K. Colwell, and A. M. Ellison. 2014. Rarefaction and extrapolation with Hill numbers: A framework for sampling and estimation in species diversity studies. *Ecological Monographs* 84: 45–67. doi:10.1890/13-0133.1.

Chicco, D., and G. Jurman. 2020. The advantages of the Matthews correlation coefficient (MCC) over F1 score and accuracy in binary classification evaluation. *BMC Genomics* 21. BMC Genomics: 1–13. doi:10.1186/s12864-019-6413-7.

Granger, C. J. W. 1969. Investigating Causal Relations by Econometric Models and Cross-spectral Methods. *Econometrica* 37: 424–438.

Granger, C. W. J. 1980. Testing for causality. A personal viewpoint. *Journal of Economic Dynamics and Control* 2: 329–352. doi:10.1016/0165-1889(80)90069-X.
